# An overview of the multi-pronged approach in the diagnosis of Alport syndrome for 22 children in Northeast China

**DOI:** 10.1186/s12882-020-01962-y

**Published:** 2020-07-23

**Authors:** Li Zhang, Bai-chao Sun, Bing-gang Zhao, Qing-shan Ma

**Affiliations:** Department of Pediatric Nephrology, First Hospital, Jilin University, Changchun, 130021 Jilin China

**Keywords:** Alport syndrome, Children, Multi-pronged approach for diagnosis, Therapy

## Abstract

**Background:**

Alport syndrome (AS) is a kind of progressive hereditary nephritis induced by mutations of different genes that encode collagen IV. The affected individuals usually develop hematuria during childhood, accompanying with gradual deterioration of renal functions. In this study, the multi-pronged approach was employed to improve the diagnosis of AS.

**Methods:**

Twenty-two children were diagnosed and treated at the Department of Pediatric Nephrology of Jilin University First Hospital between January 2017 and January 2020 using the multi-pronged approach. The following information was collected from patients, including age of onset, age at diagnosis, clinical manifestations, family history, renal pathology and genotype.

**Results:**

All these 22 children were diagnosed with Alport syndrome according to the diagnostic criteria formulated by the Japanese Society of Nephrology (2015), among them, only 13 children met the diagnostic criteria released in 1988. All the 22 patients presented with hematuria, and proteinuria to varying degrees was observed in some patients. Three children suffered from hearing loss, but no child in the cohort had any visual problem or renal failure. Meanwhile, five patients were estimated to be at Stage 2, whereas the remaining 17 cases were at Stage 0. Renal biopsies were performed in 18 patients, including 14 showing glomerular basement membranes (GBM)-specific abnormalities. Moreover, 13 children were detected with mutations of genes encoding collagen IV.

**Conclusions:**

The multi-pronged approach helps to improve the diagnosis of AS. Most patients do not have renal failure during childhood, but close assessment and monitoring are necessary. Also, the advancements in treatment are reviewed.

## Background

Alport syndrome (AS) is a hereditary disease related to type IV collagen, which usually results in progressive renal fibrosis and end-stage renal disease (ESRD) [1]. AS arises from mutations in genes *COL4A3*, *COL4A4*, and *COL4A5*. Typically, the X-linked AS (XL-AS), which is induced by mutations in the gene encoding the α5 chain of *COL4 (COL4A5)* on chromosome X, is the most common. According to previous study, the frequencies of XL-AS, autosomal recessive AS (ARAS), and autosomal dominant AS (ADAS) are estimated to be 80–85, 15%, and 1–5%, respectively [2]. Recent reports suggest that, around 60% AS patients belong to the XL-AS, while ARAS accounts for about 15% and ADAS occupies 25% [3, 4]. However, many patients harboring heterozygous mutations of *COL4A3* or *COL4A4* gene remain undiagnosed due to the subclinical course of disease and incomplete penetrance, as a result, it is difficult to determine the accurate prevalence [5].

In addition to hematuria and progressive renal failure, the affected patients also frequently suffer from extrarenal illnesses involving ears (sensorineural deafness) and eyes (peri-macular flecks and lenticonus) [6]. With the advancements in medical technology, apart from electron microscopy [7], other investigation modes, such as immunohistochemical (IHC) analysis on basement membrane type IV collagen expression through skin or renal biopsy and genetic testing [8], have broadened the clinical and research repertoire to detect the changes in AS. Typically, the commonly used diagnostic criteria include family history (FH) of hematuria, sensorineural hearing loss, characteristic eye signs, diffuse esophageal leiomyomatosis, ultrastructural changes and abnormal distributions of the α(IV) collagen chains detected by IHC staining of glomerular basement membranes (GBM), and mutations of *COL4A3*, *COL4A4* or *COL4A5* gene [9]. Nonetheless, some clinicians can still not detect this disease due to insufficient assessment or atypical presentations. At the same time, not all patients can afford the fees of all examinations. This study aimed to review the problem that, some cases were missed due to the excessively stringent criteria for AS diagnosis. To address this problem, this study tested the hypothesis that implementing the more relaxed criteria similar to those defined by the Japanese Society of Nephrology improved the diagnosis of AS cases among our patients. In addition, the risk of progression was estimated and the progress in treatment was reviewed.

## Methods

### Diagnostic criteria

In order to improve the diagnosis of AS, the criteria established by the Working Group for Alport Syndrome in the Japanese Society of Pediatric Nephrology (JSPN) in 2015 was adopted (Table 1) [10]. Meanwhile, the diagnostic criteria formulated in 1988 were also listed [11].

**Table 1:** JSPN diagnostic criteria^a^ [10] and criteria described in 1988^b^ [11]

	Diagnostic features of Alport syndrome
**I. Primary feature:**	I-1. Persistent hematuria
**II. Secondary features:**	II-1. Mutations in type IV collagen genes
	II-2. Type IV collagen abnormal expression
	II-3. Glomerular basement membrane (GBM) -specific abnormalities
**III. Accessory features**	III-1. Family history of kidney diseases
	III-2. Bilateral sensorineural deafness
	III-3. Ocular abnormalities
	III-4. Diffuse leiomyomatosis

^a^1. Diagnostic criteria: In addition to the primary feature, patients should satisfy one or more secondary features or satisfy two or more of the accessory features. 2. If patients only have the primary feature and a family member diagnosed with Alport syndrome, the case is set as a “suspected case”. 3. If patients have any one feature of type IV collagen (II-1 or II-2) among the secondary features, the case is set as “asymptomatic carriers”. 4. Features caused by other diseases should be excluded, for example, a family history of kidney failure due to diabetes

^b^Diagnostic criteria: If the proband and other family members between them meet at least three of the following:

1. Positive FH of macro/microscopic hematuria or chronic renal failure

2. Electron microscopic evidence of AS on renal biopsy

3. Characteristic ophthalmic signs (anterior lenticonus and macular flecks)

4. High-tone sensorineural deafness

### Risk evaluation criteria

In this study, three criteria (Tables 2, 3 and 4) [6, 12, 13] were adopted to estimate the risk of renal progression, including clinical estimate and genotype-phenotype correlation in XL-AS.

**Table 2:** Stages in the development of Alport syndrome [6]

**Stage 0**	microscopic hematuria (< 30 mg albumin per g creatinine or per day)
**Stage 1**	microalbuminuria (30–300 mg albumin per g creatinine or per day)
**Stage 2**	gross proteinuria (> 300 mg albumin per g creatinine or per day)
**Stage 3**	impaired renal function (GFR < 60 ml/min/1.73m^2^)
**Stage 4**	end-stage renal disease

*GFR* glomerular filtration rate

**Table 3:** New classifification system for Alport syndrome and related disorders [12]

Inheritance	Affected gene(s)	Genetic state	Comments	Estimated risk of ESRD
X-linked	COL4A5	Hemizygous (male subjects)	Rate of progression to ESRD and timing of extrarenal manifestations strongly influenced by genotype	100%
		Heterozygous (female subjects)	Risk factors for progression: gross hematuria, SNHL, proteinuria, GBM thickening and lamellation	Up to 25%
Autosomal	COL4A3 or COL4A4	Recessive (homozygous or compound heterozygous)	Rate of progression to ESRD and timing of extrarenal manifestations strongly influenced by genotype	100%
		Dominant	Hematuria Includes patients previously diagnosed as TBMN/BFH Risk factors for rogression: proteinuria, FSGS, GBM thickening and lamellation, SNHL, or evidence of progression in patient or family, genetic modififiers	20% or more among those with risk factors for progression, < 1% in absence of risk factors
Digenic	COL4A3, COL4A4, and COL4A5	COL4A3 and COL4A4 mutations in *trans*	Clinical fifindings and pedigree simulate autosomal recessive transmission	Up to 100%
		COL4A3 and COL4A4 mutations in *cis*	Clinical fifindings and pedigree simulate autosomal dominant transmission	Up to 20%
		Mutations in COL4A5 and in COL4A3 or COL4A4	Inheritance pattern does not simulate any Mendelian transmission	Up to 100% (affected male subjects)

*BFH* benign familial hematuria, *ESRD* end-stage renal disease, *FSGS* focal segmental glomerulosclerosis, *GBM* glomerular basement membrane, *SNHL* sensorineural hearing loss, *TBMN* thin basement membrane nephropathy

**Table 4:** Genotype-phenotype correlation in XL-AS [13]

	Genotype	Phenotype
Type S (Severe)	large rearrangements, premature stop, frameshift, donor splice site mutations, and mutations involving the NC 1-domain, 15% de novo mutations	ESRD ~ 20 years of age, 80% hearing loss, 40% ocular lesions
Type MS (Moderate-severe)	non-glycine-X-Y missense, glycine-X-Y involving exons 21–47, in-frame and acceptor splice site mutations, 15% de novo mutations (5% de novo glycine-X-Y mutations)	ESRD ~ 26 years of age, 65% hearing loss, 30% ocular lesions
Type M (Moderate)	glycine-XY mutations involving exons 1–20, 5% de novo mutations	ESRD ~ 30 years of age, 70% hearing loss, 30% ocular lesions

### Clinical investigation

Clinical data were collected from our patients, including the age of onset, age at diagnosis, duration from onset of symptoms to diagnosis, hematuria, proteinuria, estimated glomerular filtration rate (eGFR), extrarenal symptoms and FH. Moreover, the renal histopathological findings obtained by light microscopy (LM) and electron microscopy (EM), immunofluorescence staining and IHC staining of type IV collagen were also extracted for subsequent analysis. Gene data were obtained from some children with highly suspected AS.

### Statistical analysis

The SPSS 20.0 statistical software was employed for data processing. The abnormally distributed data were expressed as medians (range), whereas the enumeration data were expressed as percentages (%).

## Results

Altogether 22 children were diagnosed with AS from January 2017 to January 2020 at the Department of Pediatric Nephrology of Jilin University First Hospital. All these patients met the diagnostic criteria, including the primary feature and at least one of the secondary features (Table 5, Table 6). The clinical characteristics, renal pathological characteristics and gene mutations of our patients were elaborated as follows. Despite of gene detection results, only 13 children were diagnosed with AS according to the 1988 criteria (Table 6).

**Table 5:** Diagnostic features of 22 Patients

Patient ID	Gender	Age at onset/diagnosis age (months)	Persistent Hematuria (I-1)	Macroscopic hematuria	Proteinuria of nephrotic range	Stage	ESRD	Mutations in type IV collagen genes (II-1)	Type IV collagen abnormal expression (II-2)	LM	EM: GBM-specific abnormalities (II-3)	FH (III-1)	Hearing loss (III-2)	Ocular changes (III-3)	Diffuse leiomyomatosis (III-4)
**P1**	**M**	**38/45**	**+**	**+**	**–**	**0**	**–**	**COL4A5**	**ND**	**ND**	**ND**	**+**	**–**	**–**	**–**
**P2**	**M**	**36/132**	**+**	**+**	**+**	**2**	**–**	**COL4A5**	**ND**	MGA	**ND**	**+**	**+**	**–**	**–**
**P3**	**M**	**108/120**	**+**	**–**	**–**	**0**	**–**	**COL4A4/COL4A4**	**intact staining for α5**	MGA	**+**	**–**	**–**	**–**	**–**
**P4**	**F**	**108/109**	**+**	**+**	**–**	**0**	**–**	**COL4A5**	**intact staining for α5**	MGA	**ND**	**+**	**–**	**–**	**–**
**P5**	**M**	**96/97**	**+**	**+**	**–**	**0**	**–**	**ND**	**ND**	MGA	**+**	**+**	**–**	**–**	**–**
**P6**	**F**	**96/97**	**+**	**+**	**–**	**0**	**–**	**COL4A5**	**intact staining for α5**	MGA	**+**	**+**	**–**	**–**	**–**
**P7**	**F**	**60/61**	**+**	**+**	**–**	**0**	**–**	**ND**	**ND**	MGA	**+**	**NA**	**–**	**–**	**–**
**P8**	**F**	**48/49**	**+**	**–**	**–**	**0**	**–**	**COL4A3**	**intact staining for α5**	MGA	**A**	**+**	**–**	**–**	**–**
**P9**	**M**	**48/84**	**+**	**+**	**+**	**2**	**–**	**COL4A5**	**ND**	**ND**	**ND**	**+**	**+**	**–**	**–**
**P10**	**F**	**60/84**	**+**	**–**	**–**	**0**	**–**	**COL4A5**	**ND**	MGA	**+**	**+**	**–**	**–**	**–**
**P11**	**F**	**24/60**	**+**	**–**	**–**	**0**	**–**	**COL4A5**	**ND**	**ND**	**ND**	**+**	**–**	**–**	**–**
**P12**	**F**	**33/34**	**+**	**+**	**–**	**0**	**–**	**COL4A5/COL4A5**	**ND**	**ND**	**ND**	**+**	**–**	**–**	**–**
**P13**	**M**	**60/61**	**+**	**+**	**–**	**0**	**–**	**COL4A3/COL4A5**	**ND**	MGA	**A**	**+**	**–**	**–**	**–**
**P14**	**M**	**72/73**	**+**	**+**	**–**	**0**	**–**	**COL4A5**	**intact staining for α5**	MGA	**+**	**+**	**–**	**–**	**–**
**P15**	**M**	**143/170**	**+**	**+**	**+**	**2**	**–**	**COL4A5**	**ND**	FSGS	**+**	**–**	**–**	**–**	**–**
**P16**	**F**	**107/108**	**+**	**+**	**–**	**0**	**–**	**ND**	**ND**	MGA	**+**	**–**	**–**	**–**	**–**
**P17**	**F**	**24/73**	**+**	**+**	**–**	**0**	**–**	**ND**	**intact staining for α5**	MGA	**+**	**–**	**–**	**–**	**–**
**P18**	**M**	**36/85**	**+**	**+**	**–**	**0**	**–**	**ND**	**loss of staining for α5**	MGA	**+**	**+**	**–**	**–**	**–**
**P19**	**M**	**24/121**	**+**	**+**	**+**	**2**	**–**	**ND**	**loss of staining for α5**	MsPGN	**+**	**+**	**+**	**–**	**–**
**P20**	**M**	**59/61**	**+**	**+**	**+**	**2**	**–**	**ND**	**intact staining for α5**	MGA	**+**	**+**	**–**	**–**	**–**
**P21**	**F**	**59/81**	**+**	**+**	**–**	**0**	**–**	**ND**	**discontinuous for α5**	MGA	**+**	**–**	**–**	**–**	**–**
**P22**	**M**	**118/122**	**+**	**+**	**–**	**0**	**–**	**ND**	**intact staining for α5**	MGA	**+**	**+**	**–**	**–**	**–**
**Total**	**22**		**22**	**18**	**5**		**0**	**13**	**3**		**14**	**16**	**3**	**0**	**0**

*M* male, *F* female, *ESRD* end-stage renal disease, *LM* light microscope, *EM* electron microscope, *MGA* minor glomerular abnormalities, *FSGS* focal segmental glomerulosclerosis, *MsPGN* mesangial proliferative glomerulonephritis, *GBM* glomerula basement membrane, *FH* family history, *ND* Not do, *NA* not available, *A* atypical

**Table 6:** Whether one patient meets the diagnostic criteria or not?

Patient ID	JSPN in 2015 [10]	Criteria described in 1988 [11]
**P1**	**Meet: (I-1) + (II-1) + (III-1)**	**Meet: Mutations + FH**
**P2**	**Meet: (I-1) + (II-1) + (III-1) + (III-2)**	**Meet: Mutations + FH + High-tone sensorineural deafness**
**P3**	**Meet: (I-1) + (II-1) + (II-3)**	**Meet: Mutations + EM**
**P4**	**Meet: (I-1) + (II-1) + (III-1)**	**Meet: Mutations + FH**
**P5**	**Meet: (I-1) + (II-3) + (III-1)**	**Not meet**
**P6**	**Meet: (I-1) + (II-1) + (II-3) + (III-1)**	**Meet: Mutations + FH + EM**
**P7**	**Meet: (I-1) + (II-3)**	**Not meet**
**P8**	**Meet: (I-1) + (II-1) + (III-1)**	**Not meet**
**P9**	**Meet: (I-1) + (II-1) + (III-1) + (III-2)**	**Meet: Mutations + FH + High-tone sensorineural deafness**
**P10**	**Meet: (I-1) + (II-1) + (II-3) + (III-1)**	**Meet: Mutations + FH + EM**
**P11**	**Meet: (I-1) + (II-1) + (III-1)**	**Meet: Mutations + FH**
**P12**	**Meet: (I-1) + (II-1) + (III-1)**	**Meet: Mutations + FH**
**P13**	**Meet: (I-1) + (II-1) + (III-1)**	**Meet: Mutations + FH**
**P14**	**Meet: (I-1) + (II-1) + (II-3) + (III-1)**	**Meet: Mutations + FH + EM**
**P15**	**Meet: (I-1) + (II-1) + (II-3)**	**Meet: Mutations + EM**
**P16**	**Meet: (I-1) + (II-3)**	**Not meet**
**P17**	**Meet: (I-1) + (II-3)**	**Not meet**
**P18**	**Meet: (I-1) + (II-2) + (II-3) + (III-1)**	**Not meet**
**P19**	**Meet: (I-1) + (II-2) + (II-3) + (III-1) + (III-2)**	**Meet: FH + EM + High-tone sensorineural deafness**
**P20**	**Meet: (I-1) + (II-3) + (III-1)**	**Not meet**
**P21**	**Meet: (I-1) + (II-2) + (II-3)**	**Not meet**
**P22**	**Meet: (I-1) + (II-3) + (III-1)**	**Not meet**
**Total**	**22**	**13**

### Clinical characteristics

The age at diagnosis among the 22 patients (including 12 boys and 10 girls) ranged from 34 to 170 (median, 84) months. Meanwhile, the duration between symptom onset and diagnosis varied from 1 to 97 (median, 5.5) months. All children (100%) had hematuria with dysmorphic red cells, among them, 18 (81.8%) had paroxysmal macroscopic hematuria in the process of upper respiratory infection. Proteinuria within the non-nephrotic range was presented in ten children (45.5%), with the proteinuria levels of less than 30 mg albumin per g creatinine or per day (Stage 0). In addition, five children (22.7%) had nephrotic range proteinuria (P2, P9, P15, P19 and P20) and were estimated to be at Stage 2. Upon diagnosis, three children (13.6%) were confirmed to have mild to moderate sensorineural hearing loss (P2, P9 and P19), even though the eGFR and vision of all children were within the normal ranges. Moreover, positive FH was identified in 16 patients (72.7%). Table 5 depicts the full details of the clinical findings.

### Renal pathological characteristics

Renal biopsies were performed in 18 children, among them, 16 (88.9%) showed minor glomerular abnormalities (MGA) through LM, while one had focal segmental glomerulosclerosis (FSGS) and one had mesangial proliferative glomerulonephritis (MsPGN), respectively. Immunoflourescence staining was negative in 6 children (33.3%), while the remaining presented with non-specific deposition of immune complex. Further, GBM-specific abnormalities were detected in 14 cases (77.8%) by EM. Two cases showed atypical results, with extensive thinning of GBM in P8 and irregular thinning of GBM in P13, respectively. Additionally, the expression levels of type IV collagen α2 and α5 chains were tested in eleven children. According to our results, all patients showed normal positive staining of GBM and tubular basement membranes for type IV collagen α2 chain. Three children (27.3%) showed abnormal expression of type IV collagen, of them, P18 and P19 (male) presented with negative staining of α5 (IV), while P21 (female) showed discontinuous α5 chain, and the remaining 8 (72.7%) exhibited intact staining for α 5 chain (Table 5).

### Gene detection

**Fig. 1:**
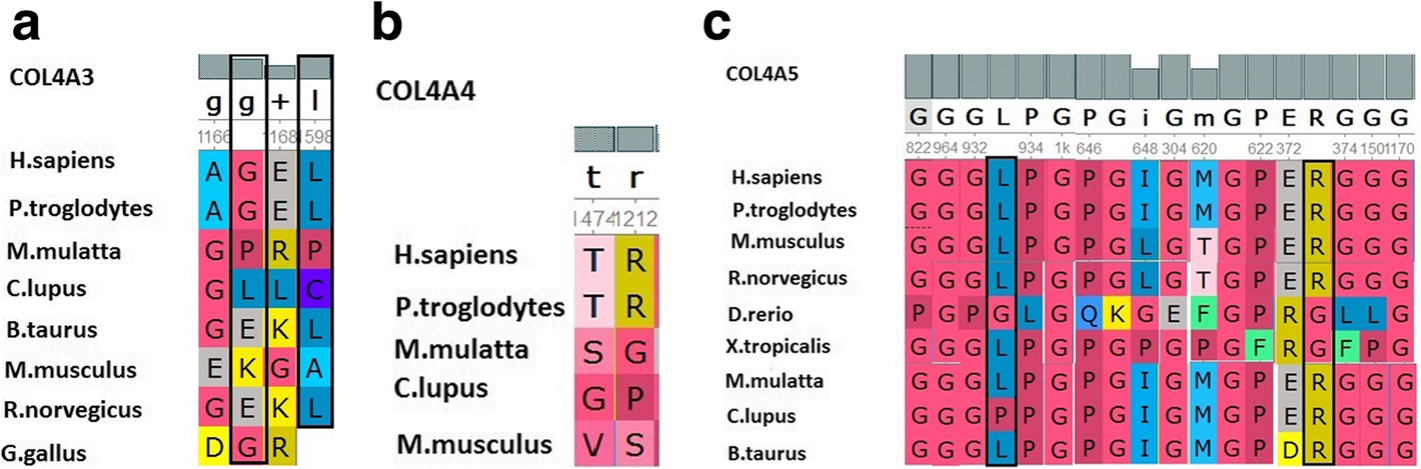
**a** Conservation of amino acid encoded by *COL4A3*. p.(Gly1167Arg) and p.(Leu1598Arg) are conserved in similar species. **b** Conservation of amino acid encoded by *COL4A4*. p.(Thr1474Met) and p.(Arg1212fs) are conserved in similar species. **c** Conservation of amino acid encoded by *COL4A5*. p.(Gly822Glu), p.(Gly964X), p.(Leu933Phe), p.(Gly1000Val), p.(Gly647Arg), p.(Gly304Glu), p.(Gly621Ala), p.(Gly374X), p.(Arg373Gln), p.(Gly150Arg), p.(Gly1170Ser) and p.(Gly150Trp) are conserved in similar species

Thirteen children and their parents were tested by the high throughput-targeted next generation sequencing (NGS) technologies at the Beijing Zhiyin Oriental Transforming Medical Research Center Co., Ltd., Beijing Jinzhun Gene Science or Centre of Genetic Diagnosis of Jilin University First Hospital. Whole exome sequencing (WES) was accepted in most patients, while “Panel” was adopted in some patients. The protein conservation was validated by the UGENE software and shown in Fig. 1a-c. Meanwhile, the pathogenicity was predicted by the online Polyphen2 and SIFT software.

Table 7 depicts the detailed mutations of genes encoding type IV collagen. Among them, ten mutations (76.9%) were inherited in the X linked manner (8 from maternal side, 1 from paternal side, 1 of indeterminacy). Additionally, five boys harbored the *COL4A5* missense mutation, including one (P2) with premature stop (Type S), three (P1, P9 and P14) with glycine-X-Y substitutions involving exons 21–47 (Type MS), and one (P15) with glycine-XY substitutions involving exons 1–20 (Type M). In the meantime, five girls had *COL4A5* mutation, including one (P4) with non-glycine-X-Y missense mutation (Type MS), two (P6 and P11) with glycine-X-Y substitutions involving exons 21–47 (Type MS), one (P10) with glycine-XY substitutions involving exons 1–20 (Type M), and one (P12) with compound heterozygous mutations of *COL4A5*, where one sequence variant led to premature stop (Type S). Besides, P3 was identified with compound heterozygous mutations of *COL4A4*, P8 with autosomal dominant mutation of *COL4A3*, and P13 with both sequence variants of *COL4A3* and *COL4A5*.

**Table 7:** Mutation of gene in 13 children with AS

ID	Gene	Location of chr	Exon	Nucleotide Change	Amino acid Change	Conservation of the protein	Source of variation	ACMG	Mutation type	Inheritance	Type
**P1**	*COL4A5*	chrX:107858210	exon30	c.[2465G>A]	p.(Gly822Glu)	conserved	Mo(het)	VUS	Missense	XL	MS
**P2**	*COL4A5*	chrX:107866028	exon33	c.[2890G>T]	p.(Gly964X)	conserved	Mo(het)	Likely pathogenic	Nonsense	XL	S
**P3**	***COL4A4***	chr2:227875130	exon46	c.[4421C>T]	p.(Thr1474Met)	conserved	**Mo(het)**	VUS	**Missense**	AR	**/**
	*COL4A4*	chr2:227896933–227,896,934	exon39	c.[3636_3637del]	p.(Arg1212fs)	conserved	**Fa(het)**	Likely pathogenic	**Frame shift**	AR	**/**
**P4**	*COL4A5*	chrX:107865935	exon33	c.[2797C>T]	p.(Leu933Phe)	conserved	Mo(het)	VUS	Missense	XL	MS
**P6**	*COL4A5*	chrX:107867547	exon34	c.[2999G>T]	p.(Gly1000Val)	conserved	Mo(het)	Pathogenic	Missense	XL	MS
**P8**	***COL4A3***	chr2:228159760	exon40	c.[3499G>A]	p.(Gly1167Arg)	conserved	Mo(het)	Pathogenic(PS1 + PM1 + PM2 + PM + PP1)	Missense	**AD**	/
**P9**	*COL4A5*	chrX:107842091	exon25	c.[1939G>A]	p.(Gly647Arg)	conserved	Mo(het)	Likely pathogenic	Missense	XL	MS
**P10**	*COL4A5*	chrX:107824232	exon16	c.[911G>A]	p.(Gly304Glu)	conserved	**Fa(hemi)**	Likely pathogenic (PM1 + PM2 + PM + PP1)	Missense	XL	M
**P11**	*COL4A5*	chrX:107842014	exon25	c.[1862G>A]	p.(Gly621Ala)	conserved	Mo(het)	Likely pathogenic (PS + PM1 + PM5)	Missense	XL	MS
**P12**	*COL4A5*	chrX:107829932	exon19	c.[1120G > T]	p.(Gly374X)	conserved	**indeterminacy**	Likely pathogenic	Nonsense	XL	S
	*COL4A5*	chrX-107,829,930	exon19	c.[1118G > A]	p.(Arg373Gln)	conserved	**indeterminacy**	VUS	Missense	XL	MS
**P13**	***COL4A3***	chr2–228,175,529	exon51	c.[4793 T > G]	p.(Leu1598Arg)	conserved	Mo(het)	Likely pathogenic	Missense	**A**	/
	*COL4A5*	chrX:107815050	exon8	c.[448G > C]	p.(Gly150Arg)	conserved	Mo(het)	VUS	Missense	XL	M
**P14**	*COL4A5*	chrX:107909779	exon39	c.[3508G > A]	p.(Gly1170Ser)	conserved	Mo(het)	Likely pathogenic (PS1 + PM2 + PP3 + PP1)	Missense	XL	MS
**P15**	*COL4A5*	chrX:107815050	exon8	c.[448G > T]	p.(Gly150Trp)	conserved	**indeterminacy**	Likely pathogenic (PS2 + PM2 + PP3)	Missense	XL	M

*Mo* mother, *Fa* father, *XL* X-linked, *AR* autosomal recessive, *AD* autosomal dominant, *ACMG* The American College of Medical Genetics and Genomics, *Het* heterozygous, *hemi* hemizygote, *VUS* uncertain significance

## Discussion

It has been well documented that, mutations of genes encoding type IV collagen leads to AS. Studies investigating the correlations of mutations with genotype and phenotype have been under way in China [14, 15]. However, as a developing country, there are still many economically less-developed regions in China, especially in Northeast China. Due to economic reasons, WES can not be extensively accepted in all families, while renal biopsy is not widely accepted for conservative idea as well. Given the above reasons, many patients can not get adequate assessment. Without sufficient evidence, the diagnosis of AS can be ambiguous and the treatment may be delayed. Therefore, the Japanese diagnostic criteria [10] were applied in this study to diagnose this disease by the multi-pronged approach, hoping to reduce the misdiagnosis rate and improper treatment, estimate the risk of progressive renal disease, provide timely intervention, and minimize the economic costs.

Using the JSPN diagnostic criteria, 22 children were diagnosed with AS. In addition to the primary feature, patients should satisfy at least one secondary feature or at least two accessory features. All patients (100%) in our cohort generally presented with persistent hematuria, which was identified as the primary feature in the criteria, and proteinuria was also detected in some patients. This was consistent with published articles [16]. As mentioned above, not all patients received NGS or renal biopsy. Only five patients (P3, P6, P10, P14, P15) received both NGS and renal biopsy, which showed positive results. In addition, six patients with positive FH, including four (P1, P9, P11 and P12) who did not receive renal biopsy and two (P2, P4) who refused to perform renal histopathology in EM, were confirmed with *COL4A5* variants by NGS. Besides, P8 showed diffuse thinning of GBM, whereas P13 showed irregular thinning of GBM. Although they were not typical in EM, they were confirmed by WES. In the meantime, they also had positive FH. The remaining nine patients were confirmed with AS due to the typical GBM abnormalities. According to the criteria, these patients satisfied the primary feature, at least one secondary feature, accompanying with or without at least one accessory feature. Although some of these patients did not receive renal biopsy, while some did not undergo genetic test, the diagnosis was credible. When it came to the original criteria, only 13 of our children were diagnosed with AS. In comparison with the previous general diagnostic criteria, the Japanese criteria improved our diagnosis and covered patients who were easy to be ignored or ambiguous.

Not all of our patients were correctly diagnosed with AS at presentation. The median duration between symptom onset and diagnosis was 5.5 (range, 1–97) months, suggesting that it took nearly half a year to get diagnosed since onset. For patients who were willing to receive necessary examminations, only 1 month was required to identify the etiology. However, much longer time was needed for more patients, even 4–8 years. In that case, many patients might receive improper treatments with an ambiguous diagnosis.

**Fig. 2:**
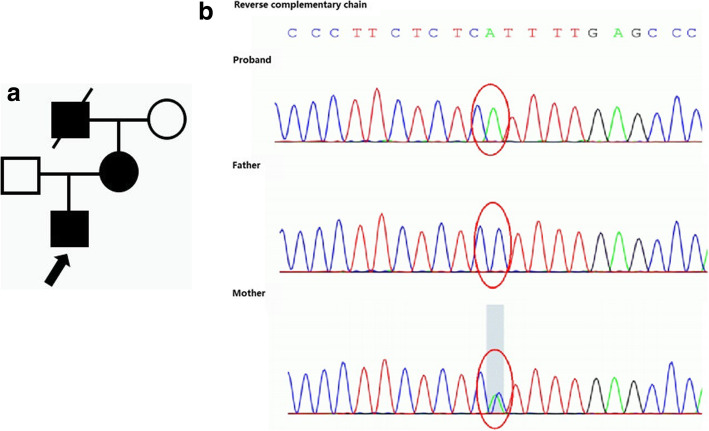
**a** The family pedigree of P2. The proband’s mother has persistent hematuria and proteinuria with unknown cause and his grandfather died of uremia. **b** Genomic analysis. WES results of the patient and his parents indicate that P2 has one variant in the *COL4A5* gene: c.[2890G>T], p.(G964X), exon33, chrX:107866028, inherited from his mother

P2 was initially diagnosed with glomerulonephritis at the age of 3 years in the local clinic due to hematuria and nephrotic range of proteinuria. Subsequent renal biopsy, which was done when he was 9 years old, was compatible with minimal change disease (MCD). Further FH revealed that his mother had persistent hematuria and proteinuria of unknown etiology and that his grandfather died of uremia, his parents refused to undergo further investigation (Fig. 2a). The patient was initially managed with corticosteroids, followed by cyclophosphamide and mycophenolate mofetil, due to steroid resistance. After Tacrolimus treatment, the patient achieved partial remission, with urine protein being controlled at below 1 g per day. The patient was later confirmed with XL-AS (a missense mutation of *COL4A5* inherited from his mother) at the age of 11 years by genetic testing (A Panel of hereditary nephritis) (Fig. 2b). Meanwhile, he was detected with hearing loss. He is currently treated with tacrolimus and ACE inhibitors. According to the Japanese criteria, the boy should be classified as a “suspected case” long before he was diagnosed. Therefore, the timing of renal biopsy or re-biopsy or genetic testing is important for those “suspected cases”. Likewise, P9, P15, P19 and P20 presented with heavy proteinuria that might have been treated as nephrotic syndrome if no further examination was performed. Interestingly, P9 and P15 were finally treated with tacrolimus after genetic confirmation of AS, and their proteinuria levels reduced to below 1 g per day. This phenomenon corroborates those previous studies showing the therapeutic benefits of calcineurin inhibitors for AS patients [17].

**Fig. 3:**
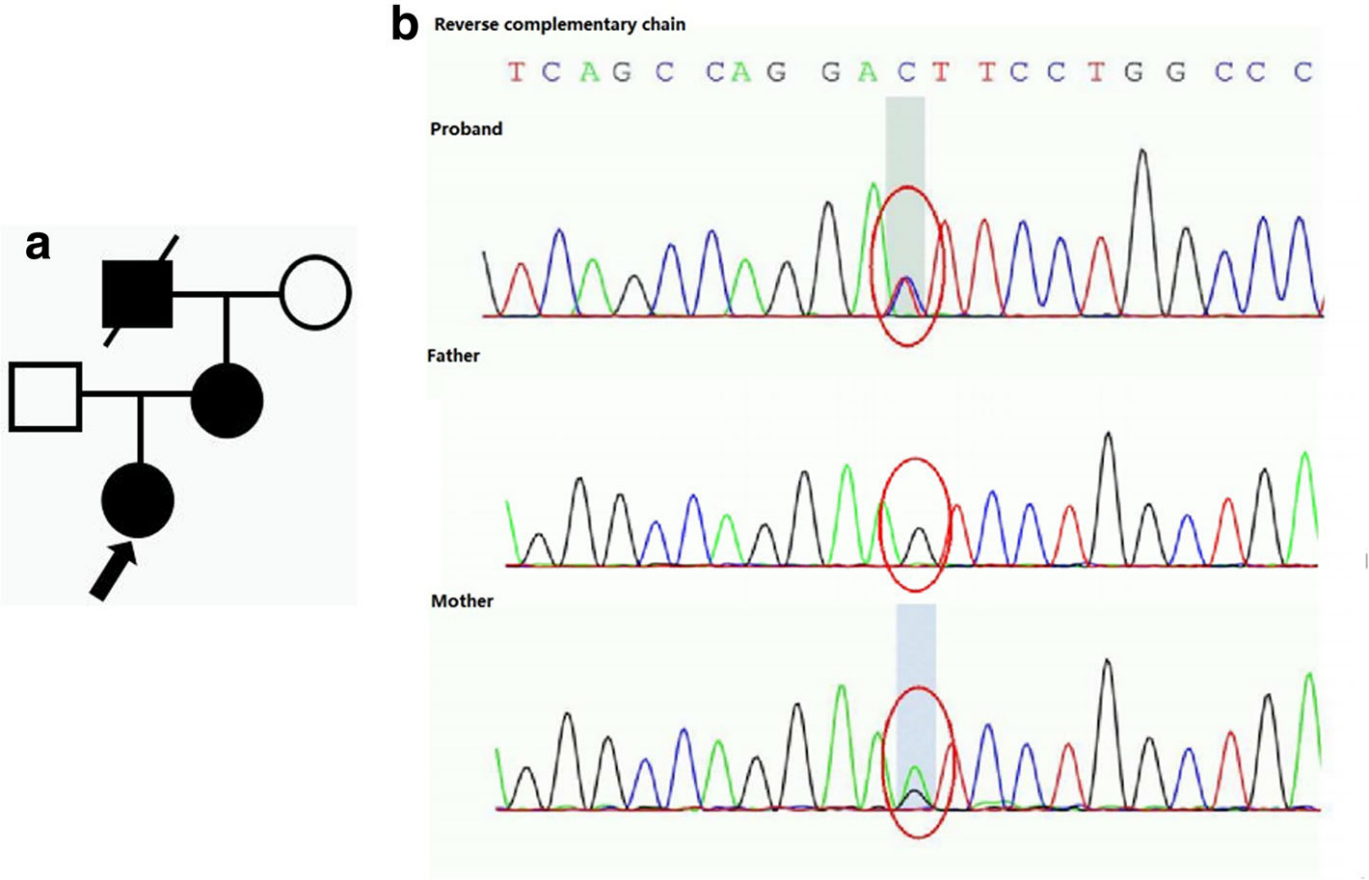
**a** The family pedigree of P4. The proband’s mother has persistent hematuria and her grandfather died of uremia. **b** Genomic analysis. WES results of the patient and her parents indicate that P4 has one variant in the *COL4A5* gene: c.[2797C > T], p.(L933F), exon33, chrX:107865935, inherited from her mother

The clinical manifestations of AS can occasionally be confused with other clinical entities. For instance, P4 of our cohort, who had a missense mutation of *COL4A5* inherited from her mother (Fig. 3b), presented with macroscopic hematuria during infection, and she did not suffer from any hearing loss or visual problem. The renal biopsy of this patient revealed MGA, along with mild IgA deposition that was compatible with IgA nephropathy. If not for the presence of FH (Fig. 3a) that made her as the “suspected case”, the patient would have been treated as IgA nephropathy, accordingly, genetic testing was not performed. Interestingly, a child with similar clinical manifestations to our patient was misdiagnosed with IgA nephropathy [18]. His renal biopsy did not reveal features of AS until he had a second renal biopsy at 4 years later. Similarly, in another recent Chinese report [19], the proband who presented with hematuria and proteinuria was initially diagnosed with IgAN by renal biopsy and immunofluorescence detection. Because of the poor treatment outcome, the patient was identified with a novel mutation of *COL4A5* by the gene detection. By the time a definite diagnosis was made, the patient had been treated with prednisolone accompanied with mycophenolate mofetil and tacrolimus successively.

**Fig. 4:**
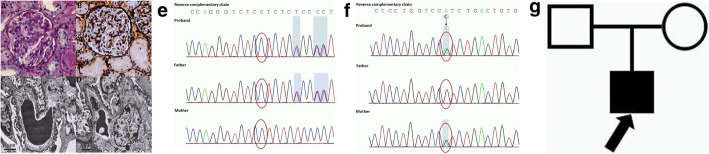
The renal pathology of P3. **a, b** LM shows minor glomerular abnormalities in PAS and PASM staining. **c, d** EM shows lamellation in GBM and fusion of the podocytic process. Genomic analysis. WES results of the patient and his parents indicate that P3 has 2 variants in the *COL4A4* gene: **e** c.[3636_3637del], p.(R1212fs), exon39, chr2:227896933–227,896,934, NM_000092, inherited from his father; **f** c.[4421C>T], p.(T1474M), exon46, chr2:227875130, NM_000092, inherited from his mother. The family pedigree of P3. **g** Both of his parents are with normal phenotype

Different from the typical manifestations, P3 displayed isolated hematuria with negative FH. The patient accidentally discovered microscopic hematuria in a health check. During the eight-month follow-up period, the urine red blood cell count fluctuated from 10/HPF to 30/HPF. GBM-specific abnormalities were observed in renal biopsy. Besides, WES revealed compound heterozygous mutations of *COL4A4*. The renal pathology, gene mutation and family pedigree of P3 are shown in Fig. 4a-g.

**Fig. 5:**
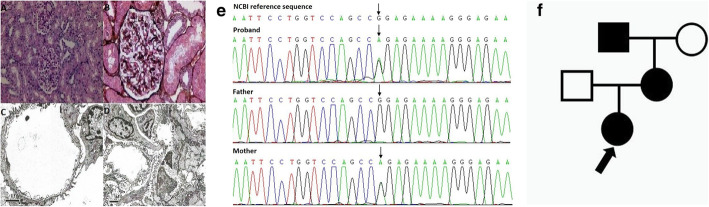
The renal pathology of P8. **a, b** LM shows minor glomerular abnormalities in PAS and PASM staining. **c, d** EM shows extensive thinning of GBM (70–150 nm) and fusion of the podocytic process. Genomic analysis. **e** WES results of the patient and her parents indicate that P8 has one variant in the *COL4A3* gene: c.[3499G>A], p.(G1167R), exon 40, chr2:228159760, NM_000091, inherited from her mother. The family pedigree of P8. **f** Her mother presented with asymptomatic hematuria, while her grandfather received regular dialysis for 8 years since the diagnosis of uremia

**Fig. 6:**
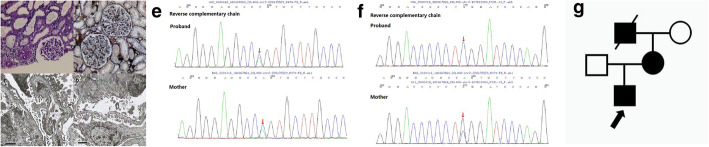
The renal pathology of P13. **a, b** LM shows minor glomerular abnormalities in PAS and PASM staining. **c, d** EM shows irregular thinning of GBM. Genomic analysis. WES results of the patient and his parents indicate that P13 has 2 variants: **e** c.[4793 T > G], p.(L1598R), exon51, chr2–228,175,529 in *COL4A3*, inherited from his father. **f** c.[448G > C], p.(G150R), exon8, chrX:107815050, in *COL4A5*, inherited from his mother. The family pedigree of P13. **g** The proband’s mother has persistent hematuria and proteinuria with unknown cause and his grandfather died of uremia

On the other hand, whether the characteristic changes of GBM can be present depends on multiple factors, such as the age of biopsy and different mutations. In our cohort, 14 out of the 18 renal biopsies revealed GBM-specific abnormalities, while two cases manifested as extensive thinning of GBM (P8) and irregular thinning of GBM (P13), respectively. Thin basement membrane nephropathy (TBMN) is a relatively common disease with the reported incidence rate of 1% in the general population [20]. Mutations in the *Col(IV)A3/A4* and *Col(IV)A5* coding genes may be responsible for TBMN and AS [21]. However, in children and females, the only evidence of AS may be the thinning of GBM, which can be misdiagnosed with TBMN [22]. According to the newest classification [12], TBMN is currently considered as a lesion description rather than a diagnosis. It was possible that some of our patients previously diagnosed with TBMN actually had AS. P8 had extensive thinning of GBM and would have been diagnosed with TBMN. Different from other cases, the patient got an autosomal dominant mutation of *COL4A3*. Her mother presented with asymptomatic hematuria, while her grandfather received regular dialysis for 8 years since the diagnosis of uremia. Considering the ominous outcome of AS, we hoped to diagnose her with AS rather than TBMN. The patient will be followed up for a long time and receive a second renal biopsy if necessary. The renal pathology, gene mutation and the family pedigree are shown in Fig. 5a-f. P13 had irregular thinning of GBM and digenic mutations of *COL4A3* and *COL4A5*. Meanwhile, his mother presented with persistent hematuria and proteinuria of unknown etiology, and his grandfather died of uremia. The detailed information is exhibited in Fig. 6a-g.

Type IV collagen, which is a component of the GBM, is a triple helix composed of three a chains. Specifically, the α3(IV), α4(IV) and α5(IV) chains are present in GBM, Bowman’s capsule and the basement membranes of distal and collecting tubules. In our cohort, P18 and P19 (male) showed negative staining of α5(IV), while P21 (female) showed discontinuous α5 chain, which satisfied the abnormal expression of type IV collagen. For P19, in addition to the manifestation of nephrotic range proteinuria, the patient also had hearing loss when he was diagnosed. Similarly, Samar et al. [23] stated that negative staining of α5 chain was correlated with the worse prognosis and more severe ultrastructural alterations in AS men. Additionally, eight patients had intact staining of α 5 chain. The putative causes might include the type and location of sequence variants. Hashimura et al. [24] hypothesized that, some missense and in-frame mutations of XL-AS might affect the structure of this triple helix, but its degradation rate was low. They also suggested that mutations located between exons 1 and 25 might lead to a less critical disruption of triple helix-forming process. This might explain for the positive staining in male patients with XL-AS who had milder clinical manifestations. However, the mechanism of autosomal AS has not been fully understood yet.

In addition to the definite diagnosis, the disease severity was also assessed. Five of our patients manifested with nephrotic range proteinuria and were estimated to be at Stage 2, three had hearing loss, and 14 presented with GBM-specific abnormalities by EM, but none of them showed renal failure. This might because that they were diagnosed within their first or second decades of lifetime, which gave us the time and opportunity to estimate the risk of progression and provide appropriate treatment.

In this study, a total of ten patients had *COL4A5* mutation, one had compound heterozygous mutations of *COL4A4*, one had autosomal dominant (AD) mutation of *COL4A3*, and one had digenic mutations of *COL4A3* and *COL4A5*. In XL-AS, hemizygous male patients have a 100% risk of progression to ESRD, although the progression rate and timing of extrarenal manifestations are related to the *COL4A5* genotype [12]. Heterozygous female patients have a 25% risk of progression to ESRD throughout their lifetime. But this depends on a variety of risk factors, including a history of gross hematuria in childhood, sensorineural hearing loss, proteinuria, and extensive GBM thickening and lamellation [25]. Few Gly substitutions are non-pathogenic. Gly substitutions with a charged residue, such as Arg, Glu or Asp, often result in the early-onset renal failure and more extrarenal features [26]. However, it is much more difficult to distinguish the pathogenic from the benign variants for non-Gly substitutions [27]. Our patients with *COL4A5* mutation were estimated with subtypes ranging from Type MS to Type S, and most of them had risk factors, so renal function should be monitored closely within the next decade.

Autosomal AS associated with biallelic mutations (homozygous or compound heterozygous) in *COL4A3* or *COL4A4* exhibits a recessive inheritance pattern, which is related to a 100% risk of ESRD, and the rate of progression and timing of extrarenal manifestations are affected by the genotype [12]. P3 in our study had the compound heterozygous mutations of *COL4A4* with GBM-specific renal pathology, and he was thus estimated with a 100% risk of ESRD. Patients with heterozygous mutations in *COL4A3* or *COL4A4* are considered to be affected in the presence of hematuria or proteinuria, including patients who would have been previously diagnosed with TBMN. In these individuals, the risk of ESRD is as high as 20% among those with risk factors for progression, including proteinuria, sensorineural hearing loss, FH of progression to ESRD, and renal biopsy findings of FSGS, or GBM thickening and lamellation, or all of the above. Recent systematic review states that there is a striking difference in the percentage of patients progressing to ESRD [5]. For instance, in a large cohort, many patients are misdiagnosed, since heterozygous *COL4A3*/*COL4A4* mutations (a cause of TBMN) are associated with FSGS [28]. These figures regarding the risk of ESRD are not always solid and they are dependent on different patients and diverse age ranges. In our cohort, P8 who might be diagnosed with TBMN was detected with *COL4A3* dominant mutation. Since her mother presented with isolated hematuria without ESRD, it was hopeful to look forward to the benign progression. Also, the risk of digenic inheritance should be further studied. It is reported in literature [29, 30] that, *COL4A3/A4* mutations in *cis* resemble an AD inheritance with a more severe phenotype, while *COL4A3/A4* mutations in *trans* mimicks an autosomal recessive inheritance with a less severe phenotype, and *COL4A5* combined with *COL4A3* triggers a more severe phenotype. In our cohort, P13 was a bit different. He got glycine-XY substitutions involving exons 1–20 in *COL4A5* and irregular thinning of GBM, in addition, he also got a *COL4A3* mutation, which contributed to a high risk of renal progression. These children are actively followed up and their renal progression is closely monitored at present.

There is no radical cure for AS for the time being, and attempts to use various stem cell therapies in animal models have attained ambiguous success. The use of cyclosporine, a calcineurin inhibitor, remains controversial due to its possible long-term nephrotoxic effects [17]. With the exception of cyclosporine, the renin-angiotensin-aldosterone system (RAAS) inhibitors are reported to be efficient and well tolerated to delay the progression of chronic kidney disease (CKD) in AS [31]. Gross et al. [32] carried out a double-blind, randomized, placebo-controlled, multicentre phase III trial to clarify the safety and efficacy of ramipril in child patients with AS, and discussed the efficacy of ramipril when the patients present with microhematuria only. So far, the Alport Syndrome Classification Working Group recommends to use ACEI in the presence of hematuria and overt proteinuria [12]. In addition, future therapies, including stem cells, chaperon therapy, collagen receptor blockade and anti-microRNA therapy, will shed more lights on the protection of kidneys in AS patients from further damage [33]. Through different mechanisms, therapies such as Bardoxolone, anti-miRNA-21, paricalcitol, lipid-lowering agents and epidermal growth factor receptor inhibitor, may play a certain role in mitigating renal fibrosis [34]. Meanwhile, chaperone and stem-cell based therapies are expected to show therapeutic efficacy at the collagen chains and GBM level, respectively. Nonetheless, when renal failure is inevitable, AS patients who undergo renal transplantation will have generally excellent outcomes [35]. Despite the prominent genotype-phenotype correlation, severe mutations do not impact the survival of patient or graft after transplantation [36].

## Conclusions

To sum up, given the importance of early diagnosis and economic factors, the multi-pronged approach is adopted in this study to diagnose AS and estimate the risk of progression. In condition-limited settings, it is important to follow a pragmatic approach. In addition, the Japanese criteria do improve our diagnosis. RAAS inhibitors have been testified to show safety and efficacy in delaying renal progression. Patients receiving renal transplantation have excellent outcomes, along with favorable graft survival rates. Future therapies are on the way to change the “inevitable” outcome of the disease.

## Data Availability

The datasets used and analyzed in the present study are available from the corresponding author on reasonable request.

## Abbreviations

AS: Alport syndrome
FH: Family history
GBM: Glomerular basement membrane
ESRD: End-stage renal disease
ARAS: Autosomal recessive Alport syndrome
ADAS: Autosomal dominant Alport syndrome
XL-AS: X-linked Alport syndrome
eGFR: Estimated glomerular filtration rate; LM: light microscopy
EM: Electron microscopy
MGA: Minor glomerular abnormalities
FSGS: Focal segmental glomerulosclerosis
MsPGN: Mesangial proliferative glomerulonephritis
NGS: Next generation sequencing
WES: Whole exome sequencing
TBMN: Thin basement membrane nephropathy
MCD: Minimal change disease
RAAS: Renin-Angiotensin-Aldosterone System
CKD: Chronic kidney disease
